# Treatment with MDL 72527 Ameliorated Clinical Symptoms, Retinal Ganglion Cell Loss, Optic Nerve Inflammation, and Improved Visual Acuity in an Experimental Model of Multiple Sclerosis

**DOI:** 10.3390/cells11244100

**Published:** 2022-12-16

**Authors:** Fang Liu, Moaddey Alfarhan, Leanna Baker, Nidhi Shenoy, Yini Liao, Harry O. Henry-Ojo, Payaningal R. Somanath, S. Priya Narayanan

**Affiliations:** 1 Clinical and Experimental Therapeutics, College of Pharmacy, University of Georgia, Augusta, GA 30912, USA; 2 Culver Vision Discovery Institute, Augusta University, Augusta, GA 30912, USA; 3 Charlie Norwood VA Medical Center, Augusta, GA 30904, USA; 4 Vascular Biology Center, Augusta University, Augusta, GA 30912, USA; 5 Department of Clinical Pharmacy, College of Pharmacy, Jazan University, Jazan 45142, Saudi Arabia; 6 Department of Cellular Biology and Anatomy, Augusta University, Augusta, GA 30912, USA

**Keywords:** multiple sclerosis, vision, neurodegeneration, inflammation, optic neuritis, experimental autoimmune encephalomyelitis, acrolein, spermine oxidase, MDL 72527

## Abstract

Multiple Sclerosis (MS) is a highly disabling neurological disease characterized by inflammation, neuronal damage, and demyelination. Vision impairment is one of the major clinical features of MS. Previous studies from our lab have shown that MDL 72527, a pharmacological inhibitor of spermine oxidase (SMOX), is protective against neurodegeneration and inflammation in the models of diabetic retinopathy and excitotoxicity. In the present study, utilizing the experimental autoimmune encephalomyelitis (EAE) model of MS, we determined the impact of SMOX blockade on retinal neurodegeneration and optic nerve inflammation. The increased expression of SMOX observed in EAE retinas was associated with a significant loss of retinal ganglion cells, degeneration of synaptic contacts, and reduced visual acuity. MDL 72527-treated mice exhibited markedly reduced motor deficits, improved neuronal survival, the preservation of synapses, and improved visual acuity compared to the vehicle-treated group. The EAE-induced increase in macrophage/microglia was markedly reduced by SMOX inhibition. Upregulated acrolein conjugates in the EAE retina were decreased through MDL 72527 treatment. Mechanistically, the EAE-induced ERK-STAT3 signaling was blunted by SMOX inhibition. In conclusion, our studies demonstrate the potential benefits of targeting SMOX to treat MS-mediated neuroinflammation and vision loss.

## 1. Introduction

Multiple Sclerosis (MS), a highly disabling neurological disease affecting more than 2.5 million worldwide [1,2], is characterized by inflammatory responses, neuronal damage, and demyelination [3]. MS has a heterogeneous presentation, and patients suffer from various disabilities, such as vision problems, memory loss, cognitive deficit, and movement disorders [1,4]. In addition to the motor and sensory disturbances, neuropsychiatric symptoms, such as depression and anxiety, are more common in MS than in other chronic neurological conditions [5,6]. While it is evident that neurodegeneration contributes significantly to MS pathology, the mechanisms underlying the progressive neurodegeneration remain unclear. During the last two decades, several disease-modifying therapies, including oral medications and monoclonal antibodies have been approved for treating MS [7,8,9,10,11]. While these medications have helped to manage symptoms and reduce exacerbations in MS patients, they work by exerting inhibitory or cytotoxic effects on immune cells, thereby causing side effects such as lymphopenia, cardiac dysfunction, and infection [12,13]. There is an unmet need for effective treatment with improved efficacy and safety that demands the identification of novel agents that can simultaneously target inflammation and neurodegeneration to treat MS.

Visual dysfunction in MS results from optic neuritis (ON) and is one of the most common clinical manifestations, which can lead to temporary or permanent vision loss [14,15]. ON is a condition resulting from the inflammation of the optic nerve [16]. ON is characterized by the acute development of visual field defects or visual acuity loss, with evidence of retinal ganglion cell (RGC) degeneration [17,18,19]. Experimental autoimmune encephalomyelitis (EAE) is a widely used rodent model to study MS-associated pathophysiology, including ON [20,21,22]. Earlier studies have shown that EAE mice develop retinal inflammation, RGC loss, Müller cell activation, infiltration of macrophages, and visual acuity changes [23,24,25,26]. However, the mechanisms underlying these neurodegenerative or inflammatory changes are not entirely understood.

Dysregulated polyamine metabolism has been associated with various neurodegenerative diseases [27,28]. Changes in polyamine levels have been implicated in neurological disease conditions, such as Alzheimer’s disease [29,30], Parkinson’s disease, [31] traumatic brain injury [32], and in the pathogenesis of ischemic brain damage [33,34]. Previous studies from our lab have shown the critical involvement of polyamine oxidation in mediating neuronal damage in various models of retinopathies [35,36,37,38,39]. Spermine oxidase (SMOX), a FAD-dependent enzyme, is an integral part of the polyamine interconversion cycle and is localized in the cytoplasm and nucleus of mammalian cells [40,41]. It is a key enzyme in polyamine catabolism that is essential in maintaining polyamine homeostasis [42,43]. Increasing evidence shows the involvement of SMOX in neurodegenerative diseases [44,45]. MDL 72527 (N1, N4-bis (2,3-butadienyl)-1,4-butane diamine) is a competitive inhibitor of SMOX and has demonstrated neuroprotective properties in multiple models [46,47,48]. Recent studies from our lab investigated the impact of SMOX inhibition via MDL 72527 treatment in the models of retinal excitotoxicity [37,39] and diabetic retinopathy (DR) [36,38], and indicated that SMOX inhibition is capable of suppressing retinal inflammation and reducing neuronal damage. However, the impact of SMOX inhibition in the EAE has not been investigated. In the present study, we investigated the impact of a SMOX blockade on EAE-induced retinal neurodegeneration and optic nerve inflammation

## 2. Materials and Methods

### 2.1. Animals and the Induction of EAE 

All animal procedures conducted in this study complied with the ARVO statement for the use of Animals in Ophthalmic and Vision Research. Wild-type female mice (12–13 weeks old) with C57BL/6J background were purchased from the Jackson laboratory (Jackson Laboratories, Bar Harbor, ME, USA), maintained in our animal facility, and are used in this study. The protocols used in the current study were approved by the Institutional Animal Care and Use Committee of Augusta University, Augusta, GA, USA and the Charlie Norwood VA Medical Center, Augusta, GA, USA. All efforts were taken to minimize the pain and animal suffering during the experimental procedures. Chronic EAE was induced using the Hooke EAE induction kit (Hooke laboratories, Lawrence, MA, USA, cat #EK-2110), according to the manufacturer’s instructions and our previously published method [43]. The mice were immunized on day 0 by subcutaneous injections in the flank region with an emulsion (total of 200 μL) containing myelin oligodendrocyte glycoprotein (MOG_35–55_) peptide (200 μg/mouse), along with complete Freund’s adjuvant (CFA, killed Mycobacterium tuberculosis H37Ra (final concentration 400 μg/μL)). Additionally, each mouse received 100 ng of pertussis toxin (PTX) by i.p. injection in 50 μL of PBS on day 0 (1–2 h after MOG_35–55_/CFA injection) and on day 1, post-immunization. The control groups received the immunization with CFA, without antigen (MOG peptide), along with two doses of PTX, given on day 0 and day 1.

### 2.2. Treatment with MDL 72527

A SMOX inhibitor, MDL 72527 (i.p. at a dose of 20 mg/kg in saline, three times a week), or vehicle (saline) were administered until the animals were euthanized. MDL 72527 or vehicle treatment started on day 1, post-induction. The experimental plan resulted in four groups of mice: Vehicle-treated control (Control vehicle, WT mice immunized with CFA with two injections of PTX and receiving normal saline, i.p.); Vehicle-treated EAE (Vehicle EAE, WT mice immunized with MOG peptide in CFA with two injections of PTX and receiving normal saline, i.p.); MDL 72527-treated EAE (MDL-EAE, WT mice immunized with MOG peptide in CFA with two injections of PTX, and receiving MDL 72527, i.p.); and MDL 72527-treated control (Control MDL, WT mice immunized with CFA with two injections of PTX, and receiving MDL 72527, i.p.).

### 2.3. Clinical Scoring

The clinical disease was monitored daily in a blinded fashion by measuring the progression of paralysis as per the conventional grading system: 0, no disease; 1, complete loss of tail tonicity; 2, partial hind limb paralysis (uneven gate of hind limb); 3, complete hind limb paralysis; 4, complete hind and forelimb paralysis; and 5, moribund or dead [20,43]. Animals displaying paralysis on all four limbs and/or weight loss of more than 15% were sacrificed immediately. Soft food was provided in the cage for mice showing signs of paralysis. Mice were euthanized at various time points by using an overdose of ketamine/xylazine cocktail and eyeballs or retinas/optic nerves were harvested and prepared for analysis.

### 2.4. Western Blotting

Retinas were collected and homogenized in RIPA buffer (Millipore, Burlington, MA, USA) containing protease and phosphatase inhibitors (Thermo Scientific™). Retinal protein lysates were run on SDS-PAGE and transferred to PVDF membranes (Bio-Rad, Hercules, CA, USA). (Millipore). Membranes were blocked in 5% milk (Bio-Rad, Hercules, CA, USA) in Tris-buffered saline with 0.05% Tween-20 (TBS-T) and incubated with primary antibodies (Table 1) overnight at 4 °C. The membranes were further incubated with horseradish peroxidase-conjugated secondary antibodies (Table 1). Signals were detected using the enhanced chemiluminescence (Thermo Scientific) and the ChemiDoc Imaging System (Bio-Rad, Hercules, CA, USA). Densitometry analyses were conducted using NIH ImageJ and normalized to the loading controls.

### 2.5. Immunofluorescence Staining of the Retinal and Optic Nerve Sections and Retinal Flatmounts

Immunostaining was performed on the retinal and optic nerve sections, as described previously [32,43,44]. The eyes were enucleated, fixed in 4% PFA (overnight at 4 °C), washed in PBS, and cryoprotected in 30% sucrose. The cryostat sections (10 μm) were prepared and mounted on glass slides and stored at −80 °C until used. The sections were permeabilized in 0.05% Triton X-100 (10 min) and blocked in 10% normal goat serum for 1 h at room temperature. The sections were then incubated with primary antibodies (Table 1) overnight, followed by 1 h incubation with fluorescein-conjugated secondary antibodies (Invitrogen, Carlsbad, CA, USA; 1:400), as previously described. The sections were washed in PBS and covered with mounting medium (Vector Laboratories Cat. # H-1000, Burlingame, CA, USA). Images were taken using a confocal microscope (LSM 780; Carl Zeiss, Thornwood, NY, USA).

Immunostaining of the retinal flatmounts was performed, according to the standardized methods, in our laboratory [37,49]. The eyeballs were fixed in 4% PFA at 4 °C overnight and the retinal flatmounts were permeabilized and incubated with NeuN antibody (1:200 dilution) for 2 h at 37 °C. Incubation with fluorescein-conjugated secondary antibody (Invitrogen, Carlsbad, CA, USA; 1:400) was performed overnight at 4 °C. Four images per retina were taken in the mid-periphery (500 µm from the optic nerve head) of the ganglion cell layer (GCL) using a confocal microscope (LSM 780; Carl Zeiss, Thornwood, NY, USA). NIH ImageJ software was used for the quantification of NeuN-positive cells.

### 2.6. Imaging and Quantification

Images of the retinal sections were captured using a Keyence fluorescence microscope (BZ-X800, Itasca, IL, USA) and/or confocal microscope (LSM 780; Carl Zeiss, Thornwood, NY, USA). A minimum of two sections (20 µm apart) per optic nerve or three sections per retina were utilized per animal for each antibody treatment. As described in our previous publications, [38,39], the images were acquired at 500 µm from the optic nerve head of the retinal sections and used for quantification. A minimum of two non-overlapping fields per optic nerve section were taken, resulting in a minimum of 6 images per mouse per antibody. A minimum of 5 animals per group were included in each study unless otherwise stated.

### 2.7. Hematoxylin and Eosin (H&E) Staining and Analysis of Cellular Infiltration

H&E staining was performed on the optic nerve sections at Augusta University’s histology core. Images were taken using Zeiss AxioPlan 2 microscope (Carl Zeiss, Thornwood, NY, USA) and the analysis was performed on three different non-overlapping fields of view from each optic nerve using the “point tool” function in the NIH ImageJ software (National Institutes of Health, Bethesda, MD, USA). A minimum of two sections were used from each optic nerve.

### 2.8. Optokinetic Studies

The visual acuity threshold was measured daily with the optokinetic tracking (OKT) response using Optometry software and apparatus (Cerebral Mechanics Inc., Medicine Hat, AB, Canada) as described [45,46]. Briefly, mice situated on a pedestal within a four-sided chamber were presented with vertical sine-wave gratings moving at 12°/s or gray of the same mean luminance. The grating rotation elicited reflexive tracking, which was scored via live video using a method-of-limits procedure with a yes/no criterion. Spatial resolution was taken as the asymptote of a staircase procedure. The two eyes were tested in an interleaved fashion. In situations where the mice were unsteady due to motor deficits to adequately perform OKT analysis, the measurement was not recorded for that mouse on that day.

### 2.9. Statistical Analysis

Statistical analyses were performed using GraphPad Prism 9 (GraphPad Software Inc., La Jolla, CA, USA). One-way ANOVA followed by the Tukey test was used for multiple comparisons. A *p*-value < 0.05 was considered statistically significant.

## 3. Results

### 3.1. MDL 72527 Treatment Improved EAE-Induced Motor Deficits

**Figure 1:**
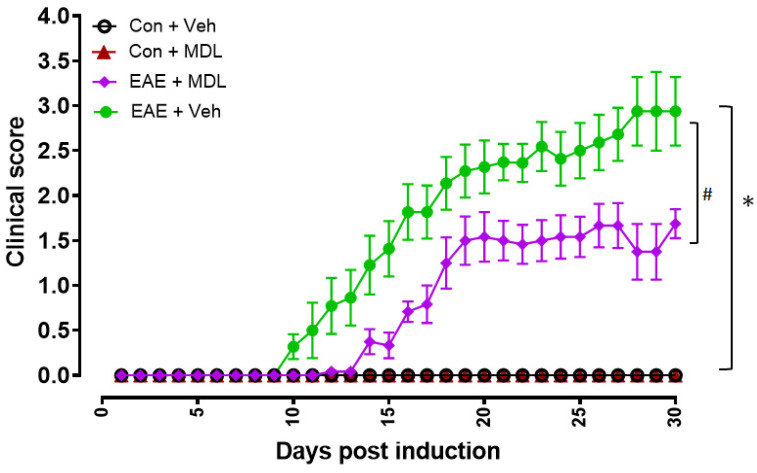
Treatment with MDL 7252 improved EAE-induced motor deficits. Animals were evaluated every day and the clinical scores were recorded according to a 0–5 scale (as described in the methods section). The mice in the vehicle-treated EAE group showed progressively increasing clinical scores starting at day 9 post-induction. EAE mice that received MDL 72527 treatments showed significantly lower clinical scores as compared to the vehicle-EAE group. Mice in both the control groups showed no signs of motor deficits. # *p* < 0.05; * *p* < 0.01; *n* = 9–15 per group. Data are presented as mean ± SEM.

In the present study, we utilized an active immunization model to induce chronic EAE in mice using a myelin oligodendrocyte glycoprotein (MOG) peptide, as published previously [49], and EAE progression was assessed up to 36 days following induction. The initial sign of paralysis in the EAE mice, indicated by the loss of tail tonicity, was observed, starting on day nine in the vehicle-treated EAE mice. Other clinical symptoms of EAE increased gradually in the EAE mice treated with vehicle, as measured by the clinical scores. As presented in Figure 1, the clinical scores were markedly reduced in EAE mice treated with MDL 72527 throughout the induction period. Interestingly, in the group of EAE mice treated with MDL, the appearance of the initial signs of paralysis was observed to be delayed (beginning at day 14) and the clinical scores were significantly lower at most of the time points, compared to the vehicle-treated EAE mice. While many EAE mice in the vehicle-treated group showed clinical scores between three and four, the scores stayed below three in most of the mice treated with MDL 72527. Those mice showing a 15% or more reduction in body weight were removed from the study and sacrificed. These results suggest that the motor deficits resulting from EAE were reduced in response to SMOX inhibition.

### 3.2. SMOX Expression Is Upregulated in the EAE Retina

**Figure 2:**
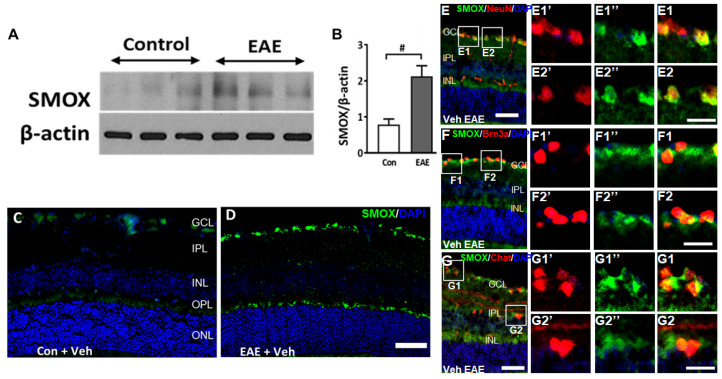
SMOX expression is upregulated in the EAE retina. (**A**,**B**) Western blot studies showing the upregulation of SMOX expression in EAE retinas compared to control, following 15 days post-induction. Quantification studies show a significantly upregulated level of SMOX in the EAE retina. Results are presented as mean ± SD; # *p* < 0.05; *n* = 4–5, representative blots are presented. (**C**,**D**) Representative immunofluorescence images showing increased expression of SMOX in the vehicle EAE retina as compared to the control. Scale bar 50 µm. (**E**–**G**) Colocalization studies showing expression of SMOX in retinal ganglion cells (Brn3a and NeuN), and amacrine cells (ChAT). Scale bar: 50 µm. Boxed areas represent areas of colocalization and magnified to visualize the expression. Red (1’ and 2’) and green (1’’ and 2”) signals are presented separately to visualize the expression pattern. E1, E2, F1, F2, G1 and G2 show magnified areas of respective colocalization. Scale bar 20 µm. GCL, ganglion cell layer; INL, inner nuclear layer; IPL, inner plexiform layer; ONL, outer nuclear layer; OPL, outer plexiform layer.

Studies were performed to assess the changes in the SMOX expression in response to EAE induction. A significant increase in the SMOX protein was observed in the retinas of vehicle-treated EAE mice (15 days post-induction) compared to the control group (Figure 2A,B). Immunofluorescence studies further confirmed the upregulation of SMOX in the EAE retinas, with higher levels in the inner retina. Localization studies performed using markers for retinal ganglion cells (Brn3a) and amacrine cells (ChAT, Choline acetyltransferase) showed expression in RGCs, colocalized with NeuN and Brn3a. SMOX expression was also observed in the outer plexiform layer (OPL) and to a lesser extent in the inner nuclear layer (INL) (Figure 2C–F).

### 3.3. SMOX Inhibition Reduced EAE-Induced Neurodegeneration

**Figure 3:**
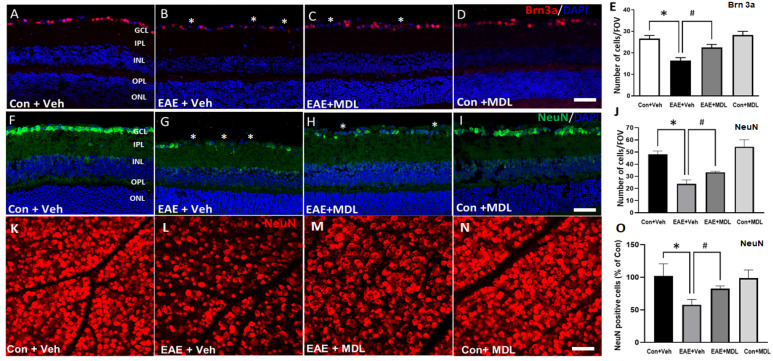
SMOX inhibition protected against RGC loss in the EAE retina. Representative confocal images showing the immunolabeling of retinal cryostat sections with Brn3a (**A**–**D**) and NeuN (**F**–**I**). Quantitative analyses demonstrated a significant loss of Brn3a-positive (**E**) and NeuN-positive (**J**) cells in the GCL in response to EAE induction. MDL 72527 treatment significantly protected against the EAE-induced RGC loss. Data are presented as mean ± SD. * (*p* < 0.01); # (*p* < 0.05). *n* = 6–7 per group. Scale bar: 50 µm. Asterisks (*) indicate areas of cell loss. Scale bar: 50 µm. GCL, ganglion cell layer; INL, inner nuclear layer; IPL, inner plexiform layer; ONL, outer nuclear layer; OPL, outer plexiform layer. (**K**–**N**): Representative confocal images of retinal flatmounts immunostained using NeuN antibody showing loss of RGCs in EAE retina and the improved protection offered by MDL 72527 treatment. Quantification study (**O**) performed using NIH ImageJ demonstrates significant loss of NeuN positive cells in Veh EAE retina and a significant survival in response to MDL 72527 treatment. Data are presented as mean ± SD. * *p* < 0.01; # *p* < 0.05. *n* = 6–7 per group. Scale bar: 50 µm.

The loss of RGCs is a major feature of EAE-induced retinal damage. In the current study, using immunofluorescence staining and analysis of the retinal sections, we investigated the EAE-induced RGC loss. Figure 3 shows the representative images of retinal sections immunostained using markers Brn3a (Figure 3A–D), and NeuN (Figure 3F–I). A significant reduction in GCL neurons is evident in the vehicle-treated EAE retinas, studied by the markers, and the treatment with SMOX inhibitor, MDL 72527, significantly improved the survival of the GCL neurons. The quantification of Brn3a (Figure 3E) and NeuN (Figure 3J) positive cells in the GCL showed significant reductions in EAE retinas compared to the vehicle-treated controls. This EAE-induced RGC loss was significantly reduced in response to MDL 72527, as measured by the markers studied. These results are further confirmed by retinal flatmount analyses using a NeuN antibody (Figure 3K–O). Compared to the vehicle-treated control group, a marked reduction in NeuN-positive cells was observed in response to EAE induction (Figure 3K,L). Treatment with MDL72527 improved the survival of NeuN-positive cells in the EAE retina (Figure 3M). Figure 3O demonstrates the quantification studies performed using NIH ImageJ. While MDL 72527 treatment offered significant RGC survival in EAE retinas, no marked changes were observed in the MDL–treated control group in response to SMOX inhibition (Figure 3O).

**Figure 4:**
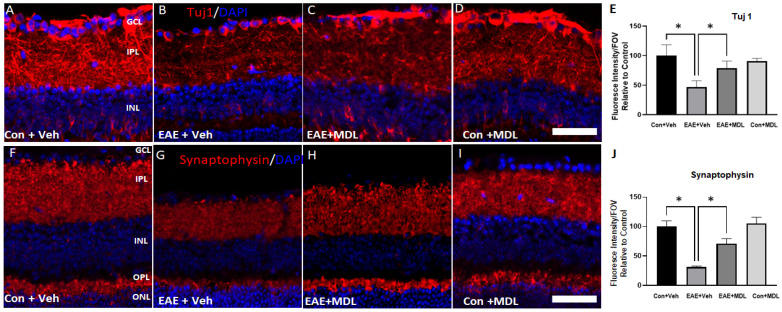
EAE-induced neurodegeneration in the inner retina is reduced by MDL 72527 treatment. Representative confocal images of retinal cryostat sections showing the immunofluorescence staining of Tuj1 (**A**–**D**), a marker for RGCs and their axons, and synaptophysin (**F**–**I**), a pre-synaptic marker. Histograms showing the ImageJ quantification of Tuj 1(**E**) and Synaptophysin (**J**) levels studied by changes in fluorescence intensity. EAE-induced downregulation of both protein markers was improved in response to SMOX inhibition by MDL 72527 treatment. Scale bar: 50 µm. Data are presented as mean ± SD. * (*p* < 0.01). GCL, ganglion cell layer; INL, inner nuclear layer; IPL, inner plexiform layer; ONL, outer nuclear layer; OPL, outer plexiform layer. *n* = 4–6 per group and representative images are presented.

Further studies using Tuj1 (Figure 4A–D) and synaptophysin (Figure 4F–I) antibodies support the EAE-induced degeneration of RGCs. A marked reduction in the Tuj1 immunostaining observed in the GCL and IPL of the vehicle-treated EAE retinas indicated axonal loss in response to EAE treatment. Our results show that MDL 72527 treatment significantly improved the levels of Tuj1 expression in the EAE retinas in comparison with the retinas from the vehicle-treated EAE mice. The quantification of Tuj1 fluorescence intensity (Figure 4E) using NIH ImageJ analysis confirmed the significantly reduced level of Tuj1 expression in the vehicle-treated EAE retina and its improved level in response to SMOX inhibition. Synaptophysin (a pre-synaptic marker) immunostaining was utilized to examine the loss of synapses due to neurodegeneration in the EAE retina. A significant reduction in synaptophysin expression was observed in the IPL and OPL of vehicle-treated EAE mice. However, MDL 72527 treatment significantly improved the levels of synaptophysin in the EAE retinas, suggesting improved synaptic contacts by SMOX inhibition. These observations are confirmed by the quantification of fluorescence intensity of the synaptophysin signal using NIH ImageJ (Figure 4J).

### 3.4. SMOX Inhibition Improved Visual Acuity in EAE Mice

**Figure 5:**
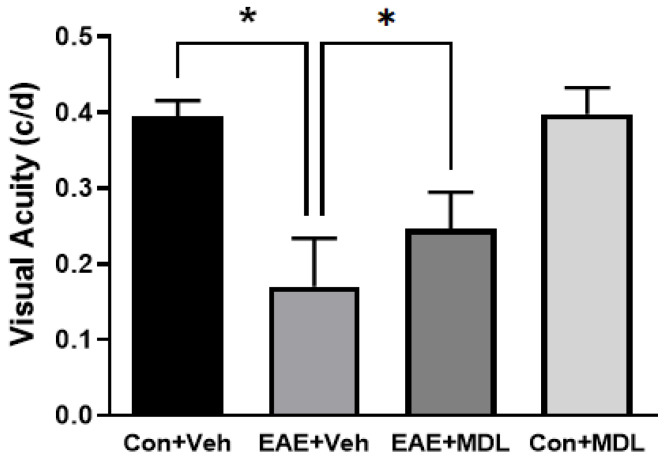
MDL 72527 improved visual acuity in EAE mice. Optokinetic studies show a reduction in visual acuity in the EAE mice (36 days post-induction) compared to the control. MDL 72527 treatment significantly improved the visual acuity. Data are presented as the average of clockwise and counterclockwise measurements. *n* = 6–10 per group; * *p* < 0.01. Data presented as mean ± SD.

In the present study, using the OptoMotry system 30 days post-induction, we investigated the impact of MDL 72527 treatment effects on visual acuity in EAE mice. Figure 5 shows the results of the optokinetic experiments utilized to study the EAE effects on visual impairment. Mice in the vehicle-treated control group showed an average response of 0.394 ± 0.02 cycles/degree (c/d). In the vehicle-treated EAE group, the average response was 0.170 ± 0.06 c/d, demonstrating a significant reduction in the OKT threshold compared to the vehicle-treated control group. However, treatment with SMOX inhibitor significantly improved the OKT threshold in the EAE mice, with 0.246 ± 0.05 c/d suggesting the improvement in vision. In comparison to the vehicle-treated control group, there was no difference in the measurements of visual acuity in the control mice treated with MDL 72527.

### 3.5. Optic Nerve Inflammation in the EAE Mice Is Reduced by SMOX Inhibition

**Figure 6:**
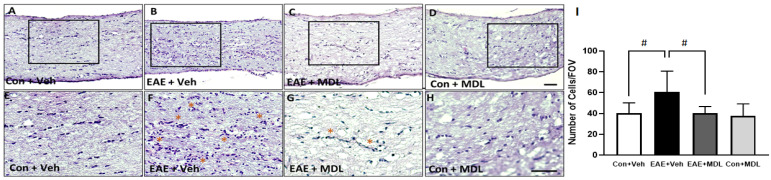
EAE-induced cellular infiltration in the optic nerve is reduced by SMOX inhibition. (**A**–**D**) Representative images of H&E stained optic nerve sections from control and EAE mice treated with vehicle or MDL 72527. Increased infiltration is evident in vehicle EAE sections, while SMOX inhibition markedly reduced this effect. (**E**–**H**) High magnification images of boxed regions demonstrate EAE-induced cellular infiltration. Clusters of infiltrated cells are shown with asterisks. (**I**) Histogram representing the quantification of infiltrated cells in optic nerve sections using ImageJ. Data are presented as mean ± SD. # (*p* < 0.05). Representative images are presented. *n* = 6–10 per group. Scale bar 50 µm.

The inflammation of the optic nerve is a major feature in EAE mice [21,50]. In the present study, we investigated the impact of MDL 72527 on EAE-induced optic nerve inflammation. Cellular infiltration was studied by the histological analysis of optic nerve sections stained by H&E. As seen in Figure 6, increased cellular infiltration is evident in the EAE optic nerves. The optic nerve sections from the vehicle-treated EAE group showed hypercellularity, compared to vehicle-treated controls (Figure 6A,B). However, in response to MDL 72527 treatment, the EAE-induced cellular infiltration was markedly reduced in the optic nerve samples (Figure 6C). The high magnification images (Figure 6E–H) collected from the optic nerve sections show clusters of infiltrated cells in the vehicle-treated EAE optic nerves compared to the MDL 72527 treated EAE and the vehicle-treated control optic nerve samples. Figure 6I presents the quantification of the infiltrated cells, demonstrating a two-fold increase in the vehicle-treated EAE optic nerve, compared to the vehicle-treated control. The EAE optic nerves from MDL 72527 treated mice showed significantly decreased levels of cellular infiltration, indicating the reduced optic nerve inflammation offered by SMOX inhibition.

**Figure 7:**
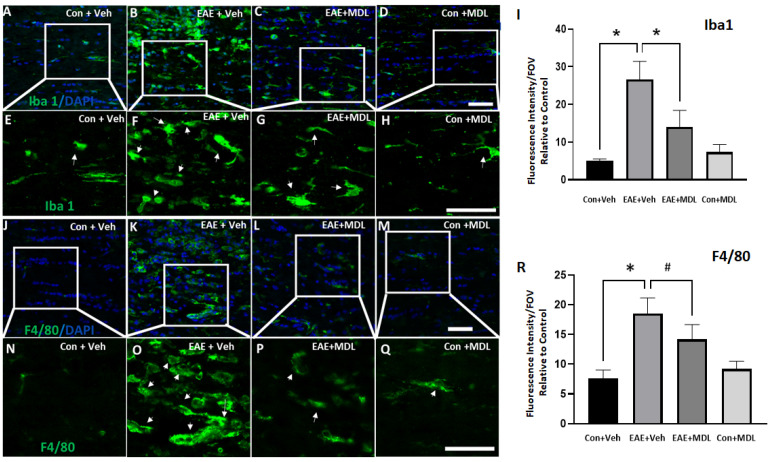
SMOX inhibition reduced the EAE-induced microglia/macrophage activation in the optic nerve. (**A**–**D**) Confocal images of Iba1 and immunofluorescence staining of optic nerve sections show increases in cells with activated morphology in response to EAE induction and the impact of SMOX inhibition by treatment with MDL 72527. (**E**–**H**) Magnified images of the boxed regions demonstrate changes in the morphology of Iba1 positive cells and the effects of SMOX blockade. (**I**) Quantification of fluorescence intensity of Iba1 positive cells. (**J**–**M**) Immunofluorescence staining of optic nerve sections showing the increased population of F4/80 positive cells with activated morphology in response to EAE induction, while SMOX inhibition by treatment with MDL 72527 reduced the effect. (**N**–**Q**) Magnified images of the boxed regions indicating EAE-induced changes in F4/80 positive cells and the effects of SMOX blockade. (**R**) Histogram showing the quantification of F4/80 fluorescence intensity. Arrows indicate Iba1 or F4/80 positive cells with activated morphology following EAE induction. Data are presented as Mean ± SD. * *p* < 0.01 and # *p* < 0.05. Scale bar 50 µm. *n* = 5–7 per group and representative images are presented.

We further evaluated the changes in EAE-induced optic nerve inflammation through immunofluorescence staining. Antibodies against Iba1 (Ionized calcium-binding adaptor molecule 1) and F4/80 (EGF-like module-containing mucin-like hormone receptor-like 1) were utilized to study the activation of microglia and macrophages in response to EAE (Figure 7). Resting microglia appear elongated with well-defined processes, and activated microglia show a more compact ameboid appearance in the optic nerve sections [51]. As shown in Figure 7A,B,E,F, the optic nerve sections from the vehicle-treated EAE mice demonstrated upregulation in Iba1 and F4/80 positive cells with an activated morphology compared to the vehicle-treated control group. Treatment with the SMOX inhibitor ameliorated these EAE-induced changes in the optic nerve (Figure 7C,G). These changes were evaluated by analyzing the fluorescence intensity measurements of Iba1 and F4/80 immunostaining. The quantification of the Iba1 and F4/80 expression levels (Figure 7I,R) shows a significant increase in the vehicle-treated EAE group compared with the vehicle-treated control mice, and this change was significantly reduced in the EAE group in response to MDL 72527 treatment. the optic nerve sections from the MDL 72527 treated control group did not show any noticeable alterations in the morphology of the Iba1 or F4/80 positive cells compared with the vehicle-treated control nerves.

### 3.6. Changes in Conjugated Acrolein Levels

**Figure 8:**
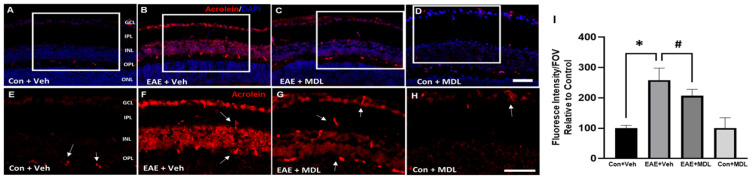
Conjugated acrolein level is downregulated in response to MDL 72527 treatment. (**A**–**D**) Representative immunostaining images of retinal cryostat sections using conjugated acrolein antibody show elevated levels of conjugated acrolein in the vehicle-treated EAE retinas (30 days post-induction) compared to control. MDL 72527 treatment markedly reduced EAE-induced acrolein formation. Arrows represent areas of nonspecific staining. Scale bar 50 µm. (**E**–**H**) Magnified images of the boxed regions demonstrating the changes in acrolein levels. (**I**) Quantification of fluorescent intensity. Data are presented as mean ± SD. * (*p* < 0.01); # (*p* < 0.05). GCL, ganglion cell layer; INL, inner nuclear layer; IPL, inner plexiform layer; ONL, outer nuclear layer; OPL, outer plexiform layer. N = 5–6 per group and representative images are presented.

Acrolein, a mediator of oxidative damage, is a major downstream effector of SMOX function [52,53]. In the present study, we employed immunofluorescence experiments to investigate the effect of the SMOX inhibitor, MDL 72527, on the changes in levels of conjugated acrolein in the EAE retina. As evident in Figure 8, an elevated level (around 3-fold) of conjugated acrolein was present in the GCL and INL of the EAE retina, while MDL 72527 treatment reduced the EAE-induced upregulation of conjugated acrolein. Quantitative studies on the fluorescence intensity on retinal sections showed a significant upregulation in the retinal samples from the vehicle-treated EAE group, compared to the vehicle-treated controls. This was significantly reduced in response to MDL 72527 treatment in the EAE retinas (Figure 8I). The presence of nonspecific staining observed in the retinal sections (as shown by arrows) was subtracted during quantification. These results support the involvement of acrolein-induced cellular damage as a potential mechanism of SMOX-regulated neurodegeneration in the EAE retina.

### 3.7. Changes in Signaling Pathways

**Figure 9:**
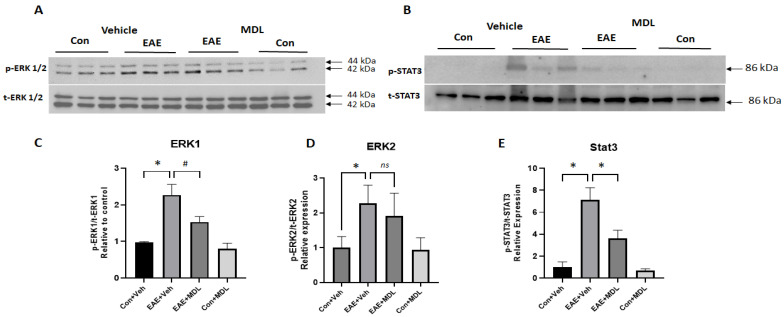
Changes in signaling pathways in the EAE retina. (**A**,**B**) Western blot studies showing upregulated levels of p-ERK1/2 and downregulation of p-STAT3 in the EAE retina. These changes are reversed in response to MDL 72527 treatment. (**C**–**E**) Quantitative analysis of western blots demonstrating increased levels of p-ERK1/2 and reduced levels of p-STAT3 in the vehicle-treated EAE retina compared to the control. SMOX inhibition altered the EAE-induced increase in p-ERK1 levels and normalized p-STAT3 levels. Data are presented as mean ± SD. * *p* < 0.01; # *p* < 0.05. *n* = 5–8 per group, and representative images are presented.

In the current study, we investigated changes in ERK1/2 and STAT3 signaling pathways in response to EAE induction. A significant increase in p-ERK1/2 was evident in the EAE retina (Figure 9A). This was accompanied by a significant upregulation of p-STAT3 (Figure 9B). Treatment with MDL 72527 reduced the level of p-ERK1, while that of p-ERK2 reduction was statistically not significant (Figure 9C,D). However, the EAE-induced increase in p-STAT3 was significantly reduced by SMOX inhibition (Figure 9E). No marked changes were observed in the status of p-ERK1/2 and p-STAT3 in the control retinas in response to MDL 72527 treatment.

## 4. Discussion

Vision impairment is one of the earliest clinical presentations of MS [15,54,55]. MS-associated ON is characterized by neurodegeneration and inflammation of the optic nerve and retina [50,56]. Current medications that are available for MS or ON target inflammation and are only partially effective [57], providing symptomatic benefits but have not been evaluated for potential long-term effects of the disease. Employing the well-established EAE model of MS, we investigated the impact of MDL 72527, a pharmacological inhibitor of the SMOX pathway, on EAE-induced neurodegeneration and optic nerve inflammation. Our results demonstrate that MDL 72527 treatment protected against EAE-induced RGC loss, the degeneration of synapses, the inflammation of the optic nerve, and visual acuity reduction. To the best of our knowledge, this is the first report demonstrating the impact of SMOX inhibition on EAE-induced retinal neurodegeneration and inflammation, suggesting the potential benefits of SMOX inhibition to treat vision loss in MS patients.

Polyamines are involved in various cellular functions and the regulation of inflammatory responses. Alterations in polyamine metabolism are shown to be involved in many neurological diseases, including MS [58,59]. Several studies have shown that the inhibition of polyamine oxidation is protective against neurodegenerative and inflammatory diseases [46,48,60,61,62]. MDL 72527 is a common inhibitor of the major polyamine oxidases (SMOX and acetyl polyamine oxidase, APAO) [63]. While APAO is constitutively expressed, SMOX is an inducible enzyme. Recent studies from our laboratory have shown the protective effects of MDL 72527 in the models of DR [38], retinal excitotoxicity [37,39], and oxygen-induced retinopathy [36]. The MOG-induced chronic EAE model has been extensively used by several laboratories to study MS and ON-associated pathologies and mechanisms [23,26,64,65]. Similar to other studies, the EAE-induced motor deficits were evident in our experimental model [23,26,66]. Further, our results demonstrated that SMOX inhibition using MDL 72527 significantly improved the clinical symptoms in EAE mice throughout the disease, with a delayed onset. These results agree with recent studies in which the supplementation of polyamines, spermine, and spermidine significantly reduced EAE-induced motor deficits [58,59]. MDL 72527 is a competitive inhibitor of polyamine oxidases, including SMOX; hence, its treatment normalizes spermine and spermidine in the pathological states. The upregulation of SMOX expression observed in the EAE retina is consistent with our findings in other retinal disease models [35,37,38]. The localization showing an expression of SMOX in the GCL, INL, and OPL in the EAE retinas is similar to what we have previously reported in OIR, diabetic, and excitotoxicity models [35,37,38]. However, in the EAE retina, RGCs showed higher expression of SMOX compared to other retinal neurons.

The loss of RGCs is a major characteristic feature of MS and EAE [20,21,67,68] and is recognized as a major cause of visual dysfunction in MS patients [69,70]. Similar to the findings from other laboratories, a significant reduction in RGCs was observed in the EAE retina in our studies. Interestingly, the EAE-induced RGC loss was reduced by MDL 72527 treatment. The quantification of RGC loss using markers such as NeuN, and Brn3a showed comparable results, which is consistent with the previously published literature on the EAE model [21,71,72]. Our results are also in support of the neuroprotection offered by MDL 72527, similar to other CNS injury models [46,47,48,61]. This was further evidenced by improved synaptic contacts and preservation of axons studied by Tuj1 and Synaptophysin immunostaining and analysis.

Several studies have indicated reduced visual acuity in the rodent EAE model [71,73,74,75]. Visual acuity changes and RGC dysfunction have also been reported in MS patients [17,76]. Improved visual acuity in EAE mice treated with MDL 72527 is an important sign of the neuroprotective efficacy of SMOX inhibition. It has been reported that the combination of retinal ganglion cell layer plus inner plexiform layer thinning is significantly correlated with both visual function and vision-specific quality of life in MS and may serve as a marker of disease activity [17]. Thus, any influence on the synaptic connections in the IPL, such as the degeneration of RGCs could be functionally important. Inflammation of the optic nerve is a major feature of MS and EAE [21,50,77], and is presented by microglia, the resident macrophages in the CNS, and other immune cells crossing the blood-brain barrier [22]. In the present study, optic nerve inflammation was characterized by increased cellular infiltration and elevated microglia/macrophages in the optic nerve, while SMOX inhibition reduced the EAE-induced optic nerve inflammation. This inflammation may lead to the demyelination of the axons. Damage to RGCs in optic neuritis has been shown to largely be a consequence of optic nerve inflammation and demyelination, leading to axonal destruction and RGC apoptosis [56,78]. Studies from MS patients and EAE models have shown that neuronal death may occur before, and independent of, immune cell infiltration of the optic nerve [79,80,81,82,83]. Various theories have also been suggested in the field of MS concerning inflammation and neuronal damage in progressive MS, such as that MS-associated tissue damage is driven by inflammatory changes, inflammation in MS is secondary to neurodegeneration, and neurodegeneration and inflammation are independent events in MS pathology [84]. Further investigations are needed in this direction and learning the sequence of events will significantly contribute to treatments with more efficacy.

Several mechanisms are involved in the interaction between neurons and immune cells, leading to the disease pathology of MS. In our study, elevated levels of conjugated acrolein, a downstream effector of SMOX signaling were observed. Acrolein is capable of increasing oxidative modifications and elevating the levels of reactive oxygen species [85]. Oxidative stress-induced ROS formation has been recognized as a key component in MS pathology [86,87,88]. Recent studies have documented the role of acrolein in elevating oxidative stress in MS models [89]. Elevated levels of acrolein metabolites were observed in the urine and spinal cord samples of EAE mice [90]. These studies emphasize the crucial involvement of acrolein-mediated oxidative damage in MS pathology. However, the exact mechanisms by which acrolein is elevated in MS tissues are unknown. A recent study from our lab has shown that acrolein treatment induces the activation of microglia/macrophages in vitro [39], a major source of ROS formation in pathological conditions. In the present study, MDL 72527 treatment reduced both conjugated acrolein levels and optic nerve inflammation. Our results show that SMOX and acrolein upregulation primarily occur in retinal neurons. This suggests that the upregulation of SMOX signaling could be at least partially contributing to the inflammation, supporting the role of neurodegeneration in disease pathology. However, further studies are needed to understand how neuronal damage and inflammation are regulated. In this study, we have not investigated the status of inflammatory cells in the retina, myelination, or axonal damage. According to our previous studies, these changes are more evident during the later stages of EAE [49,50]. In our previous studies, using the excitotoxicity and DR models, the increase in acrolein-conjugated proteins was reduced by MDL 72527 treatment [38,39].

We and others have demonstrated the activation of ERK signaling and its role in neuroglial damage in the EAE model [49,59,91,92]. In the present study, EAE-mediated ERK activation is downregulated by SMOX inhibition. Further, the activation of STAT3 signaling, a downstream effector of the ERK pathway, is also regulated by MDL 72527 treatment. STAT3 signaling is demonstrated to be a major player in EAE-induced [79] tissue damage [93,94,95,96,97]. The impact of MDL 72527 indicated in the present study supports the potential of SMOX inhibition in reducing inflammation, in addition to neuroprotection. While several studies have documented the neuroprotective effects of SMOX inhibition using MDL 72527, not much information is available regarding its anti-inflammatory effects. Earlier studies from our laboratory have shown that MDL 72527 treatment reduced inflammation in models of ischemic retinopathy and excitotoxicity [36,39]. In the present study, the results show that MDL72527 does not only curb optic nerve inflammation, but in addition, it offers neuroprotection to RGCs as well. A previous study showed a reduction in H. pylori-induced inflammation in response to MDL 72527 treatment [62]. Chronic inflammation induced by Enterotoxigenic Bacteroides fragilis in C57BL/6 mice was also shown to be reduced by MDL 72527 treatment [60]. However, no studies have investigated the impact of SMOX inhibition on inflammation and neuroprotection associated with retinal diseases.

In summary, the present study highlights the impact of the SMOX blockade in reducing clinical symptoms, as well as neurodegeneration, attenuating optic nerve inflammation, and improving visual acuity, in an experimental model of MS. Therefore, targeting SMOX signaling may provide a viable new option for reducing long-term disabilities in MS patients.

## Figures and Tables

**Table 1:** List of antibodies used in the study.

Primary Antibody	Species	Dilution	Company (Catalog Number)	Technique
SMOX	Rabbit	1:200	Proteintech Group (15052-1-AP)	Immunostaining
NeuN	Rabbit	1:200	Biosensis (R-3770-100)	Immunostaining
NeuN	Mouse	1:200	Millipore, Billerica (MAB377)	Immunostaining
Brn3a	Goat	1:200	Santa Cruz Biotechnology (SC-31984)	Immunostaining
ChAT	Goat	1:200	Millipore, Billerica (AB144P)	Immunostaining
F4/80	Rat	1:200	Abcam (ab6640)	Immunostaining
Iba1	Rabbit	1:200	Fujifilm Wako Chemicals (019-19741)	Immunostaining
Synaptophysin	Mouse	1:200	Millipore Sigma (MABN1193)	Immunostaining
Tuji1	Mouse	1:200	BioLegend (801202)	Immunostaining
Acrolein	Mouse	1:200	Abcam (ab48501)	Immunostaining
Phospho STAT3	Rabbit	1:1000	Cell Signaling Technology (9145S)	Western Blot
Total STAT3	Rabbit	1:1000	Cell Signaling Technology (12640S)	Western Blot
Phospho ERK1/2	Mouse	1:2000	Cell Signaling Technology (9106S)	Western Blot
Total ERK1/2	Rabbit	1:2000	Cell Signaling Technology (4695S)	Western Blot
**Secondary Antibody**		**Dilution**	**Company (Catalog Number)**	**Technique**
Donkey anti-Rabbit IgG (H + L), Alexa Fluor 488		1:500	Invitrogen (A21206)	Immunostaining
Donkey anti-Goat IgG (H + L), Alexa Fluor 555		1:500	Invitrogen (A21432)	Immunostaining
Donkey anti-Rat IgG (H + L), Alexa Fluor 488		1:500	Invitrogen (A21208)	Immunostaining
Donkey anti-Mouse IgG (H + L), Alexa Fluor 555		1:500	Invitrogen (A31570)	Immunostaining
Goat anti-Rabbit IgG (H + L)-HRP Conjugate		1:2000	Bio-Rad (1706515)	Western Blot
Goat anti-Mouse IgG (H + L)-HRP Conjugate		1:2000	Bio-Rad (1721011)	Western Blot

## Data Availability

Not applicable.

## References

[1] Compston A., Coles A. (2008). Multiple sclerosis. Lancet.

[2] Walton C., King R., Rechtman L., Kaye W., Leray E., Marrie R.A., Robertson N., La Rocca N., Uitdehaag B., van der Mei I. (2020). Rising prevalence of multiple sclerosis worldwide: Insights from the Atlas of MS, third edition. Mult. Scler. J..

[3] Lassmann H. (2018). Multiple Sclerosis Pathology. Cold Spring Harb Perspect Med..

[4] Dendrou C.A., Fugger L., Friese M.A. (2015). Immunopathology of multiple sclerosis. Nat. Rev. Immunol..

[5] Marrie R.A., Reingold S., Cohen J., Stuve O., Trojano M., Sorensen P.S., Cutter G., Reider N. (2015). The incidence and prevalence of psychiatric disorders in multiple sclerosis: A systematic review. Mult. Scler. J..

[6] Rintala A., Matcham F., Radaelli M., Locafaro G., Simblett S., Pietro C.B.D.S., Bulgari V., Burke P., Devonshire J., Weyer J. (2019). Emotional outcomes in clinically isolated syndrome and early phase multiple sclerosis: A systematic review and meta-analysis. J. Psychosom. Res..

[7] Peterson S., Jalil A., Beard K., Kakara M., Sriwastava S. (2022). Updates on efficacy and safety outcomes of new and emerging disease modifying therapies and stem cell therapy for Multiple Sclerosis: A review. Mult. Scler. Relat. Disord..

[8] Kappos L., Bar-Or A., Cree B.A.C., Fox R.J., Giovannoni G., Gold R., Vermersch P., Arnold D.L., Arnould S., Scherz T. (2018). Siponimod versus placebo in secondary progressive multiple sclerosis (EXPAND): A double-blind, randomised, phase 3 study. Lancet.

[9] Janiec K., Wajgt A., Kondera-Anasz Z. (2001). Effect of immunosuppressive cladribine treatment on serum leucocytes system in two-year clinical trial in patients with chronic progressive multiple sclerosis. J. Pharmacol. Exp. Ther..

[10] Paty D.W., Li D., The UBC MS/MRI Study Group, The IFNB Multiple Sclerosis Study Group (1993). Interferon beta-1b is effective in relapsing-remitting multiple sclerosis: II. MRI analysis results of a multicenter, randomized, double-blind, placebo-controlled trial. Neurology.

[11] Polman C.H., O’Connor P.W., Havrdova E., Hutchinson M., Kappos L., Miller D.H., Phillips J.T., Lublin F.D., Giovannoni G., Wajgt A. (2006). A Randomized, Placebo-Controlled Trial of Natalizumab for Relapsing Multiple Sclerosis. New Engl. J. Med..

[12] Baecher-Allan C., Kaskow B.J., Weiner H.L. (2018). Multiple Sclerosis: Mechanisms and Immunotherapy. Neuron.

[13] Montalban X., Hauser S.L., Kappos L., Arnold D.L., Bar-Or A., Comi G., de Seze J., Giovannoni G., Hartung H.-P., Hemmer B. (2017). Ocrelizumab versus Placebo in Primary Progressive Multiple Sclerosis. New Engl. J. Med..

[14] Galetta S.L., Villoslada P., Levin N., Shindler K., Ishikawa H., Parr E., Cadavid D., Balcer L.J. (2015). Acute optic neuritis: Unmet clinical needs and model for new therapies. Neurol. Neuroimmunol. Neuroinflamm..

[15] Dhanapalaratnam R., Markoulli M., Krishnan A.V. (2021). Disorders of vision in multiple sclerosis. Clin. Exp. Optom..

[16] Bennett J.L., Costello F., Chen J.J., Petzold A., Biousse V., Newman N.J., Galetta S.L. (2022). Optic neuritis and autoimmune optic neuropathies: Advances in diagnosis and treatment. Lancet Neurol..

[17] Walter S.D., Ishikawa H., Galetta K.M., Sakai R.E., Feller D.J., Henderson S.B., Wilson J.A., Maguire M.G., Galetta S.L., Frohman E. (2012). Ganglion Cell Loss in Relation to Visual Disability in Multiple Sclerosis. Ophthalmology.

[18] Sakai R.E., Feller D.J., Galetta K.M., Galetta S.L., Balcer L.J. (2011). Vision in multiple sclerosis: The story, structure-function correlations, and models for neuroprotection. J. Neuroophthalmol..

[19] Fisher J.B., Jacobs D.A., Markowitz C.E., Galetta S.L., Volpe N.J., Nano-Schiavi M.L., Baier M.L., Frohman E.M., Winslow H., Frohman T.C. (2006). Relation of Visual Function to Retinal Nerve Fiber Layer Thickness in Multiple Sclerosis. Ophthalmology.

[20] Ross A.G., Chaqour B., McDougald D.S., Dine K.E., Duong T.T., Shindler R.E., Yue J., Liu T., Shindler K.S. (2022). Selective Upregulation of SIRT1 Expression in Retinal Ganglion Cells by AAV-Mediated Gene Delivery Increases Neuronal Cell Survival and Alleviates Axon Demyelination Associated with Optic Neuritis. Biomolecules.

[21] Guo J., Wang J., Guo R., Shao H., Guo L. (2022). Pterostilbene Protects the Optic Nerves and Retina in a Murine Model of Experimental Autoimmune Encephalomyelitis via Activation of SIRT1 Signaling. Neuroscience.

[22] AlRashdi B., Dawod B., Schampel A., Tacke S., Kuerten S., Marshall J.S., Côté P.D. (2019). Nav1.6 promotes inflammation and neuronal degeneration in a mouse model of multiple sclerosis. J. Neuroinflamm..

[23] Monte M.D., Cammalleri M., Locri F., Amato R., Marsili S., Rusciano D., Bagnoli P. (2018). Fatty Acids Dietary Supplements Exert Anti-Inflammatory Action and Limit Ganglion Cell Degeneration in the Retina of the EAE Mouse Model of Multiple Sclerosis. Nutrients.

[24] Nishioka C., Liang H.-F., Barsamian B., Sun S.-W. (2018). Sequential phases of RGC axonal and somatic injury in EAE mice examined using DTI and OCT. Mult. Scler. Relat. Disord..

[25] Wilmes A.T., Reinehr S., Kühn S., Pedreiturria X., Petrikowski L., Faissner S., Ayzenberg I., Stute G., Gold R., Dick H.B. (2018). Laquinimod protects the optic nerve and retina in an experimental autoimmune encephalomyelitis model. J. Neuroinflamm..

[26] Larabee C.M., Desai S., Agasing A., Georgescu C., Wren J.D., Axtell R.C., Plafker S.M. (2016). Loss of Nrf2 exacerbates the visual deficits and optic neuritis elicited by experimental autoimmune encephalomyelitis. Mol. Vis..

[27] Sharma S., Kumar P., Deshmukh R. (2018). Neuroprotective potential of spermidine against rotenone induced Parkinson’s disease in rats. Neurochem. Int..

[28] Noro T., Namekata K., Kimura A., Guo X., Azuchi Y., Harada C., Nakano T., Tsuneoka H., Harada T. (2015). Spermidine promotes retinal ganglion cell survival and optic nerve regeneration in adult mice following optic nerve injury. Cell Death Dis..

[29] Saiki S., Sasazawa Y., Fujimaki M., Kamagata K., Kaga N., Taka H., Li Y., Souma S., Hatano T., Imamichi Y. (2019). A metabolic profile of polyamines in parkinson disease: A promising biomarker. Ann. Neurol..

[30] Inoue K., Tsutsui H., Akatsu H., Hashizume Y., Matsukawa N., Yamamoto T., Toyo’oka T. (2013). Metabolic profiling of Alzheimer’s disease brains. Sci. Rep..

[31] Paik M.J., Ahn Y.H., Lee P.H., Kang H., Park C.B., Choi S., Lee G. (2010). Polyamine patterns in the cerebrospinal fluid of patients with Parkinson’s disease and multiple system atrophy. Clin. Chim. Acta.

[32] Zahedi K., Huttinger F., Morrison R., Stewart T.M., Casero R., Strauss K.I. (2010). Polyamine Catabolism Is Enhanced after Traumatic Brain Injury. J. Neurotrauma.

[33] Takano K., Ogura M., Nakamura Y., Yoneda Y. (2005). Neuronal and Glial Responses to Polyamines in the Ischemic Brain. Curr. Neurovasc. Res..

[34] Ivanova S., Batliwalla F., Mocco J., Kiss S., Huang J., Mack W., Coon A., Eaton J.W., Al-Abed Y., Gregersen P.K. (2002). Neuroprotection in cerebral ischemia by neutralization of 3-aminopropanal. Proc. Natl. Acad. Sci. USA.

[35] Narayanan S.P., Xu Z., Putluri N., Sreekumar A., Lemtalsi T., Caldwell R. (2014). Arginase 2 deficiency reduces hyperoxia-mediated retinal neurodegeneration through the regulation of polyamine metabolism. Cell Death Dis..

[36] Patel C., Xu Z., Shosha E., Xing J., Lucas R., Caldwell R., Narayanan S. (2016). Treatment with polyamine oxidase inhibitor reduces microglial activation and limits vascular injury in ischemic retinopathy. Biochim. Biophys. Acta BBA Mol. Basis Dis..

[37] Pichavaram P., Palani C.D., Patel C., Xu Z., Shosha E., Fouda A., Caldwell R., Narayanan S.P. (2019). Targeting Polyamine Oxidase to Prevent Excitotoxicity-Induced Retinal Neurodegeneration. Front. Neurosci..

[38] Liu F., Saul A.B., Pichavaram P., Xu Z., Rudraraju M., Somanath P.R., Smith S.B., Caldwell R.B., Narayanan S.P. (2020). Pharmacological Inhibition of Spermine Oxidase Reduces Neurodegeneration and Improves Retinal Function in Diabetic Mice. J. Clin. Med..

[39] Alfarhan M., Liu F., Shan S., Pichavaram P., Somanath P.R., Narayanan S.P. (2022). Pharmacological Inhibition of Spermine Oxidase Suppresses Excitotoxicity Induced Neuroinflammation in Mouse Retina. Int. J. Mol. Sci..

[40] Cervelli M., Bellini A., Bianchi M., Marcocci L., Nocera S., Polticelli F., Federico R., Amendola R., Mariottini P. (2004). Mouse spermine oxidase gene splice variants. Nuclear subcellular localization of a novel active isoform. Eur. J. Biochem..

[41] Murray-Stewart T., Wang Y., Goodwin A., Hacker A., Meeker A., Casero R.A. (2008). Nuclear localization of human spermine oxidase isoforms—Possible implications in drug response and disease etiology. FEBS J..

[42] Lechner J., O’Leary O.E., Stitt A.W. (2017). The pathology associated with diabetic retinopathy. Vis. Res..

[43] Cervelli M., Amendola R., Polticelli F., Mariottini P. (2011). Spermine oxidase: Ten years after. Amino Acids.

[44] Cervelli M., Bellavia G., D’Amelio M., Cavallucci V., Moreno S., Berger J., Nardacci R., Marcoli M., Maura G., Piacentini M. (2013). A New Transgenic Mouse Model for Studying the Neurotoxicity of Spermine Oxidase Dosage in the Response to Excitotoxic Injury. PLoS ONE.

[45] Capone C., Cervelli M., Angelucci E., Colasanti M., Macone A., Mariottini P., Persichini T. (2013). A role for spermine oxidase as a mediator of reactive oxygen species production in HIV-Tat-induced neuronal toxicity. Free Radic. Biol. Med..

[46] Dogan A., Rao A.M., Hatcher J., Rao V.L., Baskaya M.K., Dempsey R.J. (1999). Effects of MDL 72527, a specific inhibitor of polyamine oxidase, on brain edema, ischemic injury volume, and tissue polyamine levels in rats after temporary middle cerebral artery occlusion. J. Neurochem..

[47] Rao A.M., Hatcher J.F., Dogan A., Dempsey R.J. (2000). Elevated N1-Acetylspermidine Levels in Gerbil and Rat Brains After CNS Injury. J. Neurochem..

[48] Liu W., Liu R., Schreiber S.S., Baudry M. (2001). Role of polyamine metabolism in kainic acid excitotoxicity in organotypic hippocampal slice cultures. J. Neurochem..

[49] Palani C.D., Fouda A.Y., Liu F., Xu Z., Mohamed E., Giri S., Smith S.B., Caldwell R.B., Narayanan S.P. (2019). Deletion of Arginase 2 Ameliorates Retinal Neurodegeneration in a Mouse Model of Multiple Sclerosis. Mol. Neurobiol..

[50] Candadai A.A., Liu F., Fouda A.Y., Alfarhan M., Palani C.D., Xu Z., Caldwell R.B., Narayanan S.P. (2021). Deletion of arginase 2 attenuates neuroinflammation in an experimental model of optic neuritis. PLoS ONE.

[51] Davis E., Foster T., Thomas W. (1994). Cellular forms and functions of brain microglia. Brain Res. Bull..

[52] Uemura T., Takasaka T., Igarashi K., Ikegaya H. (2017). Spermine oxidase promotes bile canalicular lumen formation through acrolein production. Sci. Rep..

[53] Uemura T., Akasaka Y., Ikegaya H. (2020). Correlation of polyamines, acrolein-conjugated lysine and polyamine metabolic enzyme levels with age in human liver. Heliyon.

[54] Ford H. (2020). Clinical presentation and diagnosis of multiple sclerosis. Clin. Med..

[55] Halilovic E.A., Alimanovic I., Suljic E., Al Hassan N. (2014). Optic Neuritis as First Clinical Manifestations the Multiple Sclerosis. Mater. Socio-Med..

[56] Fairless R., Williams S.K., Hoffmann D.B., Stojic A., Hochmeister S., Schmitz F., Storch M.K., Diem R. (2012). Preclinical retinal neurodegeneration in a model of multiple sclerosis. J. Neurosci..

[57] Bennett J.L., Nickerson M., Costello F., Sergott R.C., Calkwood J.C., Galetta S.L., Balcer L.J., Markowitz C.E., Vartanian T., Morrow M. (2014). Re-evaluating the treatment of acute optic neuritis. J. Neurol. Neurosurg. Psychiatry.

[58] Yang Q., Zheng C., Cao J., Cao G., Shou P., Lin L., Velletri T., Jiang M., Chen Q., Han Y. (2016). Spermidine alleviates experimental autoimmune encephalomyelitis through inducing inhibitory macrophages. Cell Death Differ..

[59] Zheng R., Kong M., Wang S., He B., Xie X. (2022). Spermine alleviates experimental autoimmune encephalomyelitis via regulating T cell activation and differentiation. Int. Immunopharmacol..

[60] Goodwin A.C., Destefano Shields C.E., Wu S., Huso D.L., Wu X., Murray-Stewart T.R., Hacker-Prietz A., Rabizadeh S., Woster P.M., Sears C.L. (2011). Polyamine catabolism contributes to enterotoxigenic Bacteroides fragilis-induced colon tumorigenesis. Proc. Natl. Acad. Sci. USA.

[61] Doğan A., Rao A.M., Baskaya M.K., Hatcher J., Temiz C., Rao V.L.R., Dempsey R.J. (1999). Contribution of polyamine oxidase to brain injury after trauma. J. Neurosurg..

[62] Chaturvedi R., de Sablet T., Asim M., Piazuelo M.B., Barry D.P., Verriere T.G., Sierra J.C., Hardbower D.M., Delgado A.G., Schneider B.G. (2014). Increased Helicobacter pylori-associated gastric cancer risk in the Andean region of Colombia is mediated by spermine oxidase. Oncogene.

[63] Seiler N., Duranton B., Raul F. (2002). The polyamine oxidase inactivator MDL 72527. Progress in Drug Research.

[64] Niwa M., Aoki H., Hirata A., Tomita H., Green P.G., Hara A. (2016). Retinal Cell Degeneration in Animal Models. Int. J. Mol. Sci..

[65] Azuchi Y., Kimura A., Guo X., Akiyama G., Noro T., Harada C., Nishigaki A., Namekata K., Harada T. (2017). Valproic acid and ASK1 deficiency ameliorate optic neuritis and neurodegeneration in an animal model of multiple sclerosis. Neurosci. Lett..

[66] Horstmann L., Schmid H., Heinen A.P., Kurschus F.C., Dick H.B., Joachim S.C. (2013). Inflammatory demyelination induces glia alterations and ganglion cell loss in the retina of an experimental autoimmune encephalomyelitis model. J. Neuroinflamm..

[67] You Y., Barnett M.H., Yiannikas C., Parratt J., Matthews J., Graham S.L., Klistorner A. (2020). Chronic demyelination exacerbates neuroaxonal loss in patients with MS with unilateral optic neuritis. Neurol. Neuroimmunol. Neuroinflamm..

[68] Lu Q., Ganjawala T.H., Ivanova E., Cheng J.G., Troilo D., Pan Z.-H. (2016). AAV-mediated transduction and targeting of retinal bipolar cells with improved mGluR6 promoters in rodents and primates. Gene Ther..

[69] Sriram P., Wang C., Yiannikas C., Garrick R., Barnett M., Parratt J., Graham S.L., Arvind H., Klistorner A. (2014). Relationship between Optical Coherence Tomography and Electrophysiology of the Visual Pathway in Non-Optic Neuritis Eyes of Multiple Sclerosis Patients. PLoS ONE.

[70] You Y., Barnett M.H., Yiannikas C., Parratt J.D.E., Matthews J.G., Graham S.L., Klistorner A. (2021). Interferon-beta Is Less Effective Than Other Drugs in Controlling the Rate of Retinal Ganglion Cell Loss in MS. Neurol. Neuroimmunol. Neuroinflamm..

[71] Smith A.W., Rohrer B., Wheless L., Samantaray S., Ray S.K., Inoue J., Azuma M., Banik N.L. (2016). Calpain inhibition reduces structural and functional impairment of retinal ganglion cells in experimental optic neuritis. J. Neurochem..

[72] Mao P., Manczak M., Shirendeb U.P., Reddy P.H. (2013). MitoQ, a mitochondria-targeted antioxidant, delays disease progression and alleviates pathogenesis in an experimental autoimmune encephalomyelitis mouse model of multiple sclerosis. Biochim. Biophys. Acta BBA Mol. Basis Dis..

[73] Joly S., Mdzomba J.B., Rodriguez L., Morin F., Vallières L., Pernet V. (2022). B cell-dependent EAE induces visual deficits in the mouse with similarities to human autoimmune demyelinating diseases. J. Neuroinflamm..

[74] Remlinger J., Madarasz A., Guse K., Hoepner R., Bagnoud M., Meli I., Feil M., Abegg M., Linington C., Shock A. (2022). Antineonatal Fc Receptor Antibody Treatment Ameliorates MOG-IgG-Associated Experimental Autoimmune Encephalomyelitis. Neurol. Neuroimmunol. Neuroinflamm..

[75] McDougald D.S., Dine K.E., Zezulin A.U., Bennett J., Shindler K.S. (2018). SIRT1 and NRF2 Gene Transfer Mediate Distinct Neuroprotective Effects Upon Retinal Ganglion Cell Survival and Function in Experimental Optic Neuritis. Investig. Opthalmol. Vis. Sci..

[76] Barbano L., Ziccardi L., Antonelli G., Nicoletti C.G., Landi D., Mataluni G., Falsini B., Marfia G.A., Centonze D., Parisi V. (2022). Multifocal Electroretinogram Photopic Negative Response: A Reliable Paradigm to Detect Localized Retinal Ganglion Cells’ Impairment in Retrobulbar Optic Neuritis Due to Multiple Sclerosis as a Model of Retinal Neurodegeneration. Diagnostics.

[77] Sekyi M.T., Lauderdale K., Atkinson K.C., Golestany B., Karim H., Feri M., Soto J.S., Diaz C., Kim S.H., Cilluffo M. (2021). Alleviation of extensive visual pathway dysfunction by a remyelinating drug in a chronic mouse model of multiple sclerosis. Brain Pathol..

[78] Shindler K.S., Ventura E., Dutt M., Rostami A. (2008). Inflammatory demyelination induces axonal injury and retinal ganglion cell apoptosis in experimental optic neuritis. Exp. Eye Res..

[79] Dang C., Lu Y., Chen X., Li Q. (2021). Baricitinib Ameliorates Experimental Autoimmune Encephalomyelitis by Modulating the Janus Kinase/Signal Transducer and Activator of Transcription Signaling Pathway. Front. Immunol..

[80] Saidha S., Syc S.B., Ibrahim M.A., Eckstein C., Warner C.V., Farrell S.K., Oakley J.D., Durbin M.K., Meyer S.A., Balcer L.J. (2011). Primary retinal pathology in multiple sclerosis as detected by optical coherence tomography. Brain.

[81] Dembla M., Kesharwani A., Natarajan S., Fecher-Trost C., Fairless R., Williams S.K., Flockerzi V., Diem R., Schwarz K., Schmitz F. (2018). Early auto-immune targeting of photoreceptor ribbon synapses in mouse models of multiple sclerosis. EMBO Mol. Med..

[82] You Y., Graham E.C., Shen T., Yiannikas C., Parratt J., Gupta V., Barton J., Dwyer M., Barnett M.H., Fraser C.L. (2017). Progressive inner nuclear layer dysfunction in non-optic neuritis eyes in MS. Neurol. Neuroimmunol. Neuroinflamm..

[83] Hobom M., Storch M.K., Weissert R., Maier K., Radhakrishnan A., Kramer B., Bähr M., Diem R. (2004). Mechanisms and time course of neuronal degeneration in experimental autoimmune encephalomyelitis. Brain Pathol..

[84] Correale J., Marrodan M., Ysrraelit M.C. (2019). Mechanisms of Neurodegeneration and Axonal Dysfunction in Progressive Multiple Sclerosis. Biomedicines.

[85] Moghe A., Ghare S., Lamoreau B., Mohammad M., Barve S., McClain C., Joshi-Barve S. (2015). Molecular Mechanisms of Acrolein Toxicity: Relevance to Human Disease. Toxicol. Sci..

[86] Majidi-Zolbanin J., Doosti M.-H., Kosari-Nasab M., Salari A.-A. (2015). Prenatal maternal immune activation increases anxiety- and depressive-like behaviors in offspring with experimental autoimmune encephalomyelitis. Neuroscience.

[87] Castegna A., Palmieri L., Spera I., Porcelli V., Fabis-Pedrini M., Kean R., Barkhouse D., Curtis M., Hooper D. (2011). Oxidative stress and reduced glutamine synthetase activity in the absence of inflammation in the cortex of mice with experimental allergic encephalomyelitis. Neuroscience.

[88] Ravelli K.G., Santos G.D., Dos Santos N.B., Munhoz C.D., Azzi-Nogueira D., Campos A.C., Pagano R.L., Britto L.R., Hernandes M.S. (2019). Nox2-dependent neuroinflammation in an EAE model of multiple sclerosis. Transl. Neurosci..

[89] Leung G., Sun W., Zheng L., Brookes S., Tully M., Shi R. (2011). Anti-acrolein treatment improves behavioral outcome and alleviates myelin damage in experimental autoimmune encephalomyelitis mouse. Neuroscience.

[90] Tully M., Tang J., Zheng L., Acosta G., Tian R., Hayward L., Race N., Mattson D., Shi R. (2018). Systemic Acrolein Elevations in Mice With Experimental Autoimmune Encephalomyelitis and Patients With Multiple Sclerosis. Front. Neurol..

[91] Birkner K., Wasser B., Loos J., Plotnikov A., Seger R., Zipp F., Witsch E., Bittner S. (2017). The Role of ERK Signaling in Experimental Autoimmune Encephalomyelitis. Int. J. Mol. Sci..

[92] Hou H., Cao R., Quan M., Sun Y., Sun H., Zhang J., Li B., Guo L., Song X. (2018). Rapamycin and fingolimod modulate Treg/Th17 cells in experimental autoimmune encephalomyelitis by regulating the Akt-mTOR and MAPK/ERK pathways. J. Neuroimmunol..

[93] Hou H., Miao J., Cao R., Han M., Sun Y., Liu X., Guo L. (2017). Rapamycin Ameliorates Experimental Autoimmune Encephalomyelitis by Suppressing the mTOR-STAT3 Pathway. Neurochem. Res..

[94] Zou M., Chen F.-J., Deng L.-R., Han Q., Huang C.-Y., Shen S.-S., Tomlinson B., Li Y.-H. (2022). Anemoside B4 ameliorates experimental autoimmune encephalomyelitis in mice by modulating inflammatory responses and the gut microbiota. Eur. J. Pharmacol..

[95] Sun M., Liu N., Sun J., Li X., Wang H., Zhang W., Xie Q., Wang M. (2022). Curcumin regulates anti-inflammatory responses by AXL/JAK2/STAT3 signaling pathway in experimental autoimmune encephalomyelitis. Neurosci. Lett..

[96] Ling X., Wang T., Han C., Wang P., Liu X., Zheng C., Bi J., Zhou X. (2022). IFN-gamma-Primed hUCMSCs Significantly Reduced Inflammation via the Foxp3/ROR-gammat/STAT3 Signaling Pathway in an Animal Model of Multiple Sclerosis. Front. Immunol..

[97] Alhazzani K., Ahmad S.F., Al-Harbi N.O., Attia S.M., Bakheet S.A., Sarawi W., Alqarni S.A., Algahtani M., Nadeem A. (2021). Pharmacological Inhibition of STAT3 by Stattic Ameliorates Clinical Symptoms and Reduces Autoinflammation in Myeloid, Lymphoid, and Neuronal Tissue Compartments in Relapsing-Remitting Model of Experimental Autoimmune Encephalomyelitis in SJL/J Mice. Pharmaceutics.

